# Malignancy in Abdominal Wall Endometriosis: Is There a Way to Avoid It? A Systematic Review

**DOI:** 10.3390/jcm13082282

**Published:** 2024-04-15

**Authors:** Julie Alaert, Mathilde Lancelle, Marie Timmermans, Panayiotis Tanos, Michelle Nisolle, Stavros Karampelas

**Affiliations:** 1 Department of Obstetrics and Gynecology, Centre Hospitalier Universitaire Brugmann, Université Libre de Bruxelles, 1050 Brussels, Belgium; julie.alaert@ulb.be (J.A.); stavros.karampelas@chu-brugmann.be (S.K.); 2 Department of Obstetrics and Gynecology, Centre Hospitalier Universitaire Tivoli, Université Libre de Bruxelles, 7100 La Louviere, Belgium; mathilde.lancelle@ulb.be; 3 Department of Obstetrics and Gynecology, CHU of Liege—Citadelle Site, University of Liège, 4000 Liege, Belgium; marie.timmermans@chuliege.be (M.T.); michelle.nisolle@chuliege.be (M.N.)

**Keywords:** atypical endometriosis, extra-pelvic endometriosis, endometriosis-associated malignancy, abdominal wall endometriosis, abdominal scar, clear cell adenocarcinoma

## Abstract

**Background:** Malignant-associated abdominal wall endometriosis (AWE) is a rare pathology, likely to occur in 1% of scar endometriosis. The objectives of this study were to update the evidence on tumor degeneration arising from AWE to notify about the clinical characteristics, the different treatments offered to patients and their outcomes. **Methods:** A comprehensive systematic review of the literature was conducted. PubMed, Embase and Cochrane Library databases were used. Prospero (ID number: CRD42024505274). **Results:** Out of the 152 studies identified, 63 were included, which involved 73 patients. The main signs and symptoms were a palpable abdominal mass (85.2%) and cyclic pelvic pain (60.6%). The size of the mass varied between 3 and 25 cm. Mean time interval from the first operation to onset of malignant transformation was 20 years. Most common cancerous histological types were clear cell and endometrioid subtypes. Most widely accepted treatment is the surgical resection of local lesions with wide margins combined with adjuvant chemotherapy. The prognosis for endometriosis-associated malignancy in abdominal wall scars is poor, with a five-year survival rate of around 40%. High rates of relapse have been reported. **Conclusions:** Endometrial implants in the abdominal wall should be considered as preventable complications of gynecological surgeries. Special attention should be paid to women with a history of cesarean section or uterine surgery.

## 1. Introduction

Endometriosis is a common condition which is estimated to occur in about 10–15% in women of reproductive age [1]. It is defined as an inflammatory disease in which endometrial glands and stroma are atypically present in locations other than the uterus. Endometriosis can affect the ovaries, the uterosacral ligaments and the peritoneal surfaces, and less frequently, it can spread to the bladder or extra-pelvic organs (intestine, abdominal wall, thoracic cavity) [2]. The exact physiopathology of endometriosis has yet to be defined. The most accepted theory is that of retrograde menstruation, leading to endometrial cells reaching the abdominopelvic cavity via the fallopian tubes. Deep endometriosis (DE) found in extra-pelvic sites could also be explained by other theories such as the celomic metaplasia theory, the embryonic cell and lymphatic dissemination theory [3].

Most patients are asymptomatic, but in some cases, they present with a variety of non-specific symptoms, which negatively affect their quality of life [4].

Matsuo et al. (2009) report that endometriosis exhibits some common features with neoplasia. In their own words, it is characterized as an “unstrained growth with increased neovascularization; unrestrained local and distant growth, metastasis; invasion into other tissues causing tissue damage; cytological and architectural atypia; resistance to apoptosis; and similar behavior to an estrogen-dependent neoplasm” [5]. John Sampson first reported the malignant transformation of endometriosis in 1925. He proposed three criteria for the diagnosis of malignancy arising from endometriosis: (1) demonstration of benign and neoplastic endometrial tissues in the tumor, (2) the histology being compatible with endometrial origin, and (3) with no other discoverable primary tumor sites [6]. In 1953, Scott added as fourth criterion: “the morphologic demonstration of benign endometriosis being contiguous with the malignant tissue as a prerequisite for the adjunction of a malignancy originating from endometriosis” [7].

Malignant transformation is an extremely rare condition, and it is estimated to occur in 0.7 to 1.5% of all cases of endometriosis [8]. The majority (80%) of endometriosis-associated malignancies have been found in the ovary, whereas the minority (20%) are localized in extra-gonadal sites like the small or large intestine, rectovaginal septum, abdominal wall, pleura, and others [9,10]. More seldom is the malignant transformation of abdominal wall endometriosis (AWE), of which only a few cases have so far been reported. In the literature, it is suggested that endometriosis-associated malignancy arises from a transition zone as atypical endometriosis, which is an intermediate between endometriosis and malignancy. This condition is considered to have a precancerous potential [11]. Cases of regional preperitoneal lymph node involvement have been presented in the literature and the resection of regional lymph nodes has been suggested to decrease the recurrence rate of endometriosis. Low evidence data show postoperative, endometriotic cells in regional lymph nodes as a potential target of hormonal stimulation and a major source of disease recurrence. It is important to have this in mind to avoid re-operation of patients.

AWE is defined as ectopic endometrial tissue found superficially on the peritoneum of the abdominal wall. It can be primary (umbilical endometriosis) or secondary (after trauma or surgery, known as scar endometriosis) and it is mainly found in incision scars and the umbilicus [12]. The probability of developing endometriosis on an abdominal surgical scar is 0.03% to 1% [13,14]. Malignant-associated abdominal wall endometriosis is likely to occur in 1% of such cases, particularly in cesarean section scars [15]. Currently, due to its low prevalence, there is no unified treatment plan for the malignant transformation of endometriosis and lymph node involvement management. 

The primary objective of this review was to update the evidence of the literature concerning tumor degeneration resulting from AWE. A secondary objective was to notify about the clinical characteristics of endometriosis-associated malignancy in the abdominal wall, the different treatments offered to patients and their outcomes. The last objective was to demonstrate possible preventive methods. 

## 2. Materials and Methods

A systematic literature search was performed in accordance with the PRISMA 2020 checklist. The systematic review was prospectively registered with Prospero (ID number: CRD42024505274). 

Three databases, the Cochrane Library, Embase and PubMed, were searched between March 1946 and March 2023 on 23 March 2023 by the first two authors. Any questions or concerns raised were discussed with the more senior authors. A combination of the following keywords was used: “Atypical endometriosis”; “Extra-pelvic endometriosis”; “Endometriosis-associated malignancy”; “Abdominal wall endometriosis”; “cesarean section”, “Abdominal scar”; “Clear cell adenocarcinoma”. Only studies published in English and French languages were included. Studies included were required to have sufficient reporting data (including and not limited to CT, MRI, PET-CT, laparoscopy, histological evidence, and USS). Studies outside the scope of endometriosis and studies with limited reported evidence were excluded. The revised Cochrane risk-of-bias tool was utilized in studies portraying risk of bias. 

**Figure 1:**
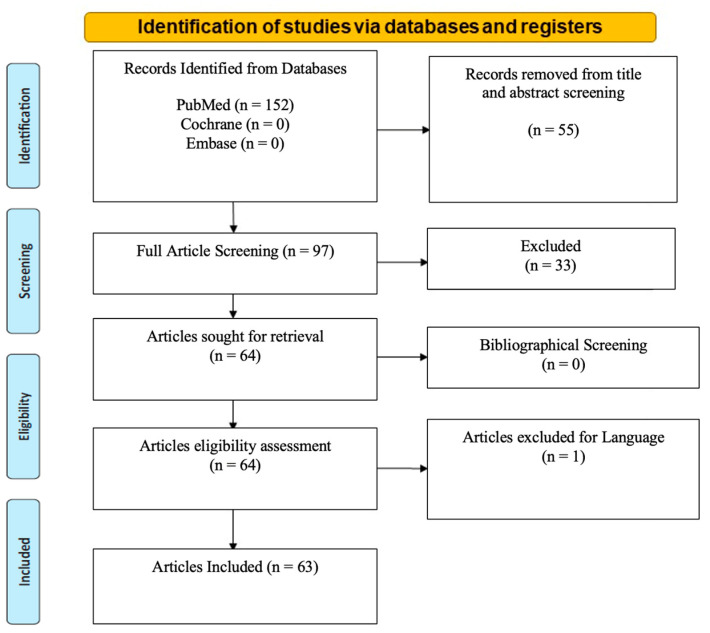
PRISMA 2020 flow diagram for new systematic reviews with included search results of databases, registers and other sources.

Over 152 studies meeting the inclusion criteria were identified. Fifty-five studies were outside the scope of malignant endometriosis and were therefore excluded. Thirty-three were excluded due to limited reported evidence, and one was excluded because of the language (Spanish). A total of 63 articles remained after elaborating bibliographical search screening. Cochrane and Embase databases did not produce any search results (Figure 1). No library filters or limits were applied. The extracted studies were exported from the medical libraries as .txt files and consecutively analyzed by being re-imported into an excel file. Data collection was carried out manually and independently with the help of three reviewers (Supplementary Table S1).

The following parameters from all eligible studies were extracted when available: lesion site, history of endometriosis, main symptoms, time interval (between operation and malignant transformation of the scar incision), pathology, lesion size, tumor marker level (CA125), type of surgery, chemotherapy, radiotherapy, follow-up and outcome (died of disease, recurrence, no evidence of disease, not available and median survival time). Median survival time was defined as the time from the date when 50% of the patients died of disease.

### Statistical Analysis

Statistical analysis was conducted in R software (version 3.6.2). Relevant summary descriptive statistics data are presented in Table 1. Comparison of background covariates between 2 groups was performed using unpaired t test for continuous variables and chi-squared test of independence or Fisher exact test (when appropriate) for categorical variables. Survival analysis was performed using the Kaplan–Meier estimates with software XLSTAT (Addinsoft, New York, NY, USA, Version 2006). *p* < 0.05 was considered significant.

## 3. Results

### 3.1. Included Studies

Seventy-three patients were included from 63 studies (Supplementary Table S1). Studies identified were mainly observational studies and case reports but there were no restrictions in the type of studies included. The inclusion criteria included all studies reporting typical or atypical endometriosis, with associated malignant transformation, at the site of surgical scaring, intra- and extra-pelvic endometriosis, abdominal scar endometriosis, women, with no age limit, any type of malignancy or associated malignancy, with and without lymph node involvement, with no year of publication limitations and with no limitations in the type of treatment and type of management or follow-up.

### 3.2. Epidemiology

The median age at the time of the diagnosis of malignant transformation of abdominal wall endometriosis was 47 years old (range 37–67 years) (Table 1).

Among the patients identified, 38 had a single history of surgery while 34 had two or more surgeries. Most patients had one cesarean section (27/38), two had laparoscopy for endometriosis, two digestive surgery, two laparoscopic hysterectomy, one had a myomectomy, one tubal ligation, two laparotomy for endometriosis and one had a left oophorectomy. When patients had at least two surgeries, the majority (23/34) had at least two cesarean sections and 16 had a history of both cesarean section and laparoscopic surgery. Only one patient had no history of surgery.

The mean interval time between first surgery and diagnosis of malignant transformation of AWE was 19.9 years. The longest interval was 41 years and the shortest 4 years.

Endometriosis is most frequently associated with ovarian endometrioid carcinoma followed by ovarian clear cell carcinoma. However, the most frequent histologic type of malignant transformation of AWE is clear cell carcinoma (52/73 patients), followed by endometrioid adenocarcinoma (11/73). Other histologic types included serous adenocarcinoma (2/73), serous papillary carcinoma (2/73), mixed endometrioid adenocarcinoma and clear cell carcinoma (4/73), mixed endometrioid and serous papillary carcinoma (1/74), and mixed endometrioid adenocarcinoma and sarcoma (1/73) (Table 2).

### 3.3. Clinical Manifestation

The primary symptoms of malignant transformation of abdominal wall included an abdominal nodule, mass, or lump (37.0%), abdominal or pelvic pain (37.0%) and/or both (19.2%) (Table 2). Other types of symptoms mentioned by patients were bleeding, ulceration and exudation. Mass size ranged from 3 to 25 cm with a median diameter of 9.4 cm.

### 3.4. Diagnostic Instruments

During the physical examination by inspection and palpation, a superficial, palpable and painful lesion was usually present. The imaging methods used for the diagnosis of the subcutaneous mass were the ultrasound (US), magnetic resonance imaging (MRI), computed tomography (CT) scan or a combination of these modalities. The ultrasonographic image varied from cystic to solid nodules with the presence of irregular borders. These boarders were located near the cesarean section scar. Biopsy via a fine needle aspiration was another modality used for diagnosis. This technique was deemed inconclusive and controversial as it could disseminate cells to the puncture site.

For the determination of the size and depth of the lesion, or its potential infiltration to the lateral tissues as well as the existence of local metastasis, CT scan and MRI were reported as useful. Positron emission tomography (PET-CT) scan was further used to evaluate systemic metastasis. Nineteen patients had only MRI (5/19) or CT scans (14/19) as part of their pre-surgical work-up. CT scan demonstrated better sensitivity and specificity than MRI (100% and 90% versus 50% and 100%, respectively) in terms of lymph node detection. The combined sensitivity and specificity of MRI and CT scans were 50% and 100%, respectively. The pre-surgery work-up sensitivity and specificity of associating MRI, CT and PET-CT scans was even higher when compared to the use of just one imaging modality.

In our study, six out of twenty-seven patients (22.2%) with a negative pre-surgery workup underwent lymph node resection, and three out of six patients (50%) had positive lymph nodes during surgery. Sixteen out of twenty patients (80%) with positive pre-surgery lymph nodes were confirmed to be positive at histopathology report and one patient was negative. Two patients had no lymph node resection, and one was lost in follow-up (Table 3).

Serum CA125 is an indirect marker to diagnose endometriosis but does not seem to be equally valuable for the diagnosis of malignant transformation of endometriosis. Out of the 73 patients, twenty-seven had a normal CA 125 level, eight had a positive but weak level and ten had a level greater than twice the normal value. There was no CA 125 screening for 28 patients (Table 2). The highest serum CA 125 level found in our review was 3157 U/mL.

### 3.5. Treatment and Follow-Up

Due to the low prevalence of the disease, there is no standard treatment plan. The most widely accepted treatment in the literature is surgical resection of local lesions with adjuvant chemotherapy (most commonly a combination of paclitaxel and carboplatin, for a mean duration of 3 to 6 months). Surgery was the first line of treatment in all patients. The primary surgical treatment was based on a local resection of the tumor with wide margins (optimal defined resection margin width in cm/mm). Any defect in the abdominal wall needed to be repaired with autologous skin and muscle flaps or with the help of a mesh. Some patients underwent endometrial curettage or hysterectomy, and/or bilateral adnexectomy and/or omentectomy, and/or lymph node resection. The surgical treatment strategy was classified into seven groups: local resection (LR), local resection and lymph node resection (LR + LNR), local resection and total hysterectomy and salpingo-oophorectomy (LR + HRT + SOT), local resection and total hysterectomy and salpingo-oophorectomy and lymph node resection (LR + HRT + SOT + LNR), local resection and total hysterectomy and salpingo-oophorectomy and omentectomy and lymph node resection (LR + HRT + SOT + omentectomy + LNR), local resection and total hysterectomy and salpingo-oophorectomy and omentectomy (LR + HRT + SOT + omentectomy) and as well as other mixed surgery treatment strategy (this includes LR with or without HRT, and LR with or without SOT).

Sixty-eight out of seventy patients (97.1%) had a local resection. LR was associated with hysterectomy in forty-three patients (61.4%) and with bilateral adnexectomy in fifty patients (71.4%). Twenty-nine of them (41.4%) underwent lymph node excision and eleven (15.7%) underwent lymph node excision and omentectomy. The extent of the lymph node excision depended on tumor localization. Surgical information was missing for one patient (Table 4). In the population analysis, only two patients did not undergo surgery and received only chemotherapy and radiotherapy. The main reasons for this decision were the extent of the disease and the poor physical condition of the patients.

However, for postoperative treatment, chemotherapy was used in most cases. Fifty-three patients (72.6%) received postoperative chemotherapy. Thirty-eight of them (71.7%) had paclitaxel and carboplatin, and the rest (23.3%) received other types of chemotherapy (platinum, gemcitabine, bevacizumab and cyclophosphamide). The mean duration of treatment was 6 months. Concomitant chemotherapy and radiotherapy were proposed to twenty-four patients (32.9%) with a postoperative recurrence.

Follow-up data were available for fifty-eight patients (79.4%). The average duration of follow-up was 22.75 months; the shortest follow-up lasted 2 months and the longest lasted 130 months. Relapse occurred in 37.9% of patients (22/58). Local relapse was found in eight cases (36.4%), lymph node recurrence in ten cases (45.4%) and four distal metastases (18.2%), of which the most frequent sites were liver, bones and lungs. Concerning lymph node recurrence, the most frequent sites were inguinal lymph nodes (6/10). Twenty-seven percent of patients with positive inguinal lymph nodes in the histopathology report had a recurrence and 27% of them died of the disease. Patients with recurrence tended to have a poorer prognosis (overall survival of 48.6 months). Complete remission was achieved in 62.1% of patients (36/58). In the postoperative histopathology result, twenty-two patients had positive lymph nodes, five of whom died of disease (22.7%). Thirty-three patients had negative lymph node invasion and four of them died of the disease (12.1%) (Table 5).

In Table 6, we compare the recurrence according to pre- and post-surgery lymph node results. No statistical difference was observed in the pre-surgery lymph node result group (*p*-value = 0.64), or in the post-surgery lymph node result group (*p*-value = 0.84). No significant statistical difference was observed in the outcome either according to pre- (*p*-value = 0.23) and post-surgery (*p*-value = 0.45) lymph node resection results or depending on the type of surgery (*p*-value = 0.82).

Among the patients followed, 13 died, which is equivalent to 22.4% of the population followed-up (13/58). The average survival was 23.3 months (ranging from 1 to 130 months). Patients who died mainly had clear cell carcinoma (9/13), and endometrioid carcinoma (2/13) or mixed (2/13).

## 4. Discussion

Endometriosis is considered as a benign condition; however, it shares certain key features with malignant tumors such as tissue invasion and destructive growth. The study by Modesitt et al. (2002) noted the presence of ‘‘transition points’’ in such tumors, where a benign endometriotic gland was observed blending with atypical and overtly malignant glands [16]. These data endorse the idea that endometriosis can undergo malignant transformation rather than simply being a coexisting diagnosis, and that atypical endometriosis can be a precancerous state [17].

As previously described by Sampson’s criteria, the coexistence of neoplastic endometrial tissue and endometriosis is pathognomonic for the diagnosis of endometriosis-associated malignancies. Ovarian and/or pelvic endometriosis is associated with approximately 42% of endometrioid ovarian cancer [18,19,20,21]. In particular, atypical endometriosis was found as the precursor of endometrioid ovarian cancer and clear cell cancer in 15–32% of the cases [20,21,22]. Pecorino et al., 2022, shows that for women with synchronous endometrial–ovarian endometrioid cancer affected by early-stage low-grade endometrioid cancer without apparent lymph node involvement at preoperative imaging have a very low rate of lymph node metastasis and similar relapse rate with or without lymphadenectomy [23]. Therefore, in endometriosis, the risk of concurrent ovarian cancer should be kept in differential diagnosis and the need for lymphadenectomy should be considered on a case-to-case basis.

However, one-third of case reports in the literature of clear cell carcinoma arising in cesarean section scars were not associated with endometriosis near the tumor [24]. Giannella et al. (2020) pointed out that in 58–64% of cases, there was an absence of a transition zone, making diagnosis even more difficult [25]. This may be explained by tumor destruction of normal tissue and endometriotic cells as they degenerate [26,27]. The clinical context may be relevant, as some patients present with cyclic pain during menstruations, which strongly suggests the presence of endometriotic implants [28,29].

### 4.1. Plausible Theories

The exact pathway of endometriosis into scar incision is not yet known. Some theories have been established over the years. Gunes et al. (2005) describe a mechanical transplantation theory in which the endometrium is accidentally transplanted into the surgical scar in patients with no history of endometriosis prior to surgical procedures. Furthermore, they note a theory of metaplasia in which scar endometriosis appears de novo [30].

Bedell et al. (2020) also distinguish two theories. The first theory is the “primary incisional carcinoma”. This theory presents established benign abdominal wall endometriosis transforming into a malignant tumor. The second theory is the “translocation theory”. According to this theory, benign endometriosis is iatrogenically transported to the surgical scar where it transforms into neoplasia. It must be emphasized that 90% of malignant scar endometriosis have undergone previous gynecological procedures with endometrium exposure, including and not limited to cesarean sections, therefore making the translocation theory more plausible [31]. This theory was first studied by Ridley et al. (1958) in their experiment with endometrial cell implantation in the abdominal wall. They showed that the endometrium is viable in vivo [32]. Marras et al. (2019) also describe the fact that patients with previous surgery had no concomitant pelvic endometriosis and presented with isolated AWE. This group was more likely to have experienced iatrogenic implantation of endometrial cells during surgery [33]. However, direct implantation of endometrial tissue cannot explain all cases of AWE. As seen in the second group of patients with concomitant pelvic endometriosis, the patients with no previous surgery developed mainly umbilical lesions [33]. Whether concomitant pelvic endometriosis plays a role in AWE is difficult to know, as currently, there are little data on pelvic laparoscopic exploration, which is not recommended. Findings from the literature indicate that there might be two different pathogenic pathways in the development of AWE and malignant AWE. Larger cohorts have shown an increased risk of cancer in women with endometriosis [18,34]. Endometriosis was significantly associated with an increased risk of clear cell, low-grade serous and endometrioid invasive cancers [35].

The typical complaint of abdominal wall endometriosis is cyclic menstrual pain and a palpable mass [36]. The differential diagnosis of a mass associated with a previous surgical incision in the abdominal wall must also include abscess, hematoma, hernia, desmoid tumor, sarcoma and metastatic disease from an ovarian, endometrial, cervical or non-gynecological neoplasia [34].

The mean time interval between the first operation and the onset of malignant transformation of abdominal wall endometriosis is 20 years, indicating that it evolves slowly towards its malignant state (between 4 and 41 years). Cesarean section is the most common obstetrical surgery in reproductive-age women. This might provide a sufficiently long period for endometriosis to undergo malignant transformation. This long interval is in favor of the translocation theory of benign endometriosis [31].

As most cases of degenerated parietal endometriosis occur after cesarean section, prevention of endometriosis implantation at the time of cesarean section seems to be important. In the literature, some instructions are given even without any scientific evidence. For example, the uterus should not be exteriorized, exposure of endometrial mucosa during uterine suturing should be limited, different instruments should be used for uterine and abdominal closure, and peritonization may be advised [37]. Some authors mentioned cases of AWE after supracervical hysterectomy, especially when no containment device was used [38]. Additionally, metastatic deposits at the port site have been reported in the literature after laparoscopy, which is a minimally invasive surgical procedure for abdominal conditions. Siddiqui et al. (2017) recommend extracting all tissues in an appropriate Endobag and deflating the pneumoperitoneum before removing the trocars [39].

In a large series of diagnostic laparoscopies in advanced stages of ovarian cancer, the incidence of port-site metastasis after surgery was studied. Vergotte et al. (2005) reported a high incidence of metastasis at the port site, despite precautionary measures [19]. Various hypothetical theories have been proposed in the literature to explain trocar port-site endometriosis as by the dissemination of endometrial cells through the pneumoperitoneum or from direct contact between the lesion and the port tract [40].

### 4.2. Risk Factors

Several risk factors for malignant-associated endometriosis need to be established, but previous gynecological surgery with endometrial exposure appears to be the most relevant. Most of the patients with primary incisional carcinoma had a surgical history (89% cesarean section and 4% myomectomies) [31]. Furthermore, the obstetric history of patients who developed scar endometriosis after cesarean section seems to be relevant. As described by Wicherek et al. (2007), cesarean sections were previously performed before the spontaneous onset of labor, with high immune tolerance, which appears to significantly increase the risk of scar endometriosis compared with cesarean sections performed during labor. It is suggested that immunological tolerance during pregnancy might facilitate the implantation of decidual cells into the surgical wound [41].

Hyperestrogenism also seems to play a role [42]. This raises the question whether estrogen therapy should be used concomitantly with a progestative agent in patients with residual endometriosis after menopause [43]. Furthermore, Tanase et al. (2019) consider the following to be risk factors for malignant endometriosis: advanced age of the patient, postmenopausal status and tumor diameter of an endometriotic lesion larger than 9 cm [44].

Currently, there is no specific tumor marker for the malignant transformation of endometriosis. Although serum CA125 it is an important indicator in advanced-stage ovarian cancer, where its value increases along with tumor size and is useful for predicting responses to chemotherapy, disease progression and recurrence, serum CA125 does not seem to be a valuable marker for the diagnosis of malignant transformation of endometriosis [18,34].

### 4.3. The Role of Pathophysiology

Even though the pathogenesis of the malignant transformation of endometriosis remains unelucidated, certain studies involved oxidative stress with epigenetic alterations in DNA methylation [1,2,3,4]. Endometriosis is also considered a chronic inflammatory process associated with immune processes. In the malignant transformation, the target to follow can be the tumor microenvironment. It is now accepted that the tumor microenvironment is essential for neoplastic development and progression. The inflammatory tumor microenvironment and its main components, non-tumoral cells (different immune cell types, fibroblasts tumor-associated), soluble factors secreted by both tumoral and non-tumoral cells, such as VEGF, FGF, EGF, IL-6, TNFα and immune checkpoint molecules, play an impact on endometriosis and tumor and malignancy development [45,46].

Immunological and biological effects which take place may cause a genomic instability and possible DNA mutations in endometriosis. Most of our knowledge so far is based on the transformation of endometriosis to ovarian cancer. Heterozygosity defect, p53 overexpression and the loss of the oncogenic K-ras PTEN may all have an effect in the transformation of endometriosis to malignancy [47]. Fibroblast growth factor (FGF-1) and interleukine 1 (IL1) are also expressed in both endometriosis and ovarian cancer, suggesting a common mechanism of action towards the malignant transformation [48].

An additional important role is played by the angiogenesis promoted by the vascular endothelial growth factor (VEGF) which can establish a local vascular network within endometriotic lesions. This effect is promoted by the hypoxic microenvironment of endometriosis, which is known to cause excessive oxidative stress. The high iron content present in endometriotic lesions may also contribute to this excessive oxidative stress, which may favor the inductions of mutations [45,49]. Yamaguchi et al. demonstrated that endometriotic cysts have an abundance of free iron that is strongly associated with frequent DNA mutations and may play a crucial role in the malignant transformation to ovarian cancers [46].

Lastly, molecular predictors of malignant transformation could exist as genetic alterations, and as a loss of heterozygosity, such as mutations in PTEN, ARID1, BAF250a and p53, which have been found in both endometriosis-associated malignancy and endometriosis [47,48].

### 4.4. The Role of Imaging

The pre-surgery work-up consists of imaging methods. Ultrasound is a non-expensive non-invasive, readily available tool to assess malignant scar endometriosis, with an appearance that can vary from cystic or nodular to solid mass. MRI helps to define the depth of extension of the mass. Blood content appears hyperintense on T1-weighted fat suppressed sequences and manifest classic “T2 shading” on T2-weighted sequences. Restricted diffusion can be seen in both benign and malignant tumors [50]. In our review, the lesions were widely described as a heterogeneous, partly solid, partly cystic mass. Only in 6/32 patients who underwent MRI was the tumor characterized by hypointense T1-weighted fat-suppressed sequences, and hyperintense on T2-weighted fat-suppressed sequences. As malignant scar endometriosis is a rare entity, there are no specific imaging characteristics in the literature.

There is evidence of iron overload, which causes severe oxidative stress and antioxidant depletion in various types of carcinomas. In MRI, the iron content of organs can be quantified by measuring the transverse magnetic relaxation rate R2. To distinguish benign ovarian endometrioma from ovarian cancer, MRI relaxometry could be a non-invasive preoperative tool (sensitivity 86%, specificity 94%) [20]. However, since the MRI models which can measure R2 values are limited and since R2 values vary between MRI devices, it is difficult to use them routinely. Kawahara et al. (2021) published a formula that estimates R2 values by incorporating CEA and tumor diameters as independent predictive factors for discriminating endometriosis ovarian cancer, from ovarian endometrioma [20,51]. Additionally, CT scan findings play a primary role in staging, as this imaging technique is more sensitive than ultrasound in detecting pelvic lymphadenopathy and is more able to assess distant thoracic metastases than MRI [52]. These findings showed that combined imaging methods, including ultrasound, MRI, CT scan and PET-CT scan, are of greater value than a single imaging method and should therefore be used during the completion of the diagnostic steps.

Novel techniques such as the ElectroUteroGraph (EUG) and computer-assisted tissue imaging analysis (CATIA) show promising results in monitoring the uterine contractility and pathologies affecting tissue histology. Further development of such techniques could be of great assistance in the early diagnosis and management of AWE [53,54].

### 4.5. Treatment

There is currently no standard treatment due to the rarity of scar endometriosis carcinoma. The only effective treatment seems to be a wide local excision with disease-free margins. The use of synthetic mesh or tissue transfer for wall closure is often necessary [47]. Zhao et al. (2005) stated that the size and depth of the infiltrating lesions in AWE are significant risk factors for recurrence. The extent of infiltrating lesions makes complete resection more challenging. For this, they suggest a 5 mm disease-free resection margin in AWE [55]. Ding et al. (2013) suggest minimum margins of 1 cm to avoid recurrence and malignant transformation, which may necessitate the resection of the fascia/muscle/peritoneum and the use of a mesh [56].

In addition to local resection, total abdominal hysterectomy or salpingo-oophorectomy was conducted in 11 of 21 cases of malignant endometriosis in abdominal wall (52.4%). However, it remains unclear whether extensive resection is necessary as no malignant lesions were observed in the uterus or in both adnexa that were resected [57]. Nevertheless, to exclude primary tumor sites and to establish the endometriotic origin, Bourdel et al. (2010) suggest radical tumor resection combined with bilateral adnexectomy and total hysterectomy or, at least, a sample of endometrial tissue collected by dilatation and curettage [58]. A total of 29 patients underwent lymph node resection. Of the 27 patients with negative pre-surgical lymph node results, six patients underwent lymph node resection and three of these six were positive. Lymph node resection was performed in most patients (17/20) with positive lymph nodes at preoperative work-up, but only seven of them had a specific resection of lymph nodes detected by diagnostic methods. The remaining patients underwent a resection of a combination of inguinal, pelvic and para-aortic lymph nodes.

Adjuvant chemotherapies were mostly taxane–carboplatin regimens, as this is the standard treatment for epithelial ovarian cancer, which was adapted to endometriosis-associated ovarian cancer [59]. PARPis (poly-ADP-ribose polymerase inhibitors) emerged as a therapeutic option in epithelial ovarian cancer with poor prognosis and recurrence. Resistance to platinum-based chemotherapy may explain the dark outcomes in malignant AWE, but it remains to be determined whether PARPis have a therapeutic place in recurrent malignant AWE [60].

In summary, primary surgery should consider wide tumor resection concomitantly with lymphadenectomy. Adjuvant chemotherapy and radiotherapy should be recommended. To clarify the multimodal treatment management of this disease, more cases are still needed.

### 4.6. Prognosis

Despite radical surgery and the multimodal treatment approach in malignant AWE, out of the 58 cases with available follow-up data, 22 relapsed and 13 died from the disease.

The small number of malignant scar endometriosis case reports and the different treatment strategies limit conclusions about prognosis. Mainly studied in malignant ovarian endometriomas, clear cell carcinoma (50% overall survival rate) seems to be more aggressive than endometrioid carcinoma (78% overall survival rate) [61]. Endometriosis-associated ovarian cancer appears to occur in younger women with a better outcome as they often present with a low-grade tumor (FIGO I or II) compared with ovarian carcinoma without endometriosis (low-grade rate: 49% versus 24%) [62]. Locally advanced cancer at the time of diagnosis can explain the poor prognosis of malignant AWE [17]. Some authors describe other prognostic factors such as the size of the mass at the time of diagnosis, which could play a role in a better outcome, especially if the masses range between 4 and 9 cm [63,64,65,66,67,68,69,70,71,72,73,74].

The 5-year survival rate calculated by Mihailovici et al. (2017) is 40% with a median survival time after diagnosis of 42 months [9]. Taburiaux et al. (2015) also demonstrated a median survival time of 30 months after diagnosis of endometriosis-associated abdominal wall cancer [17]. In this review, we noted that out of 22 patients with recurrence, eleven (50%) died of the disease with a median survival time of 12 months and only 3/11 patients (13.6%) had no evidence of disease during the follow-up. Lymphatic metastases were associated with a particularly poor prognosis. Half of the 11 patients with lymphatic metastases and available follow-up, who received adjuvant chemotherapy, died of the disease within a median of 11.5 months [31]. Additionally, in our population cohort, the patients with the presence of lymph node metastasis had a higher mortality compared to the absence of lymph node metastasis (22.7% vs. 12.1%).

### 4.7. Limitations

The limitation of this systematic review stem from the rarity of the disease. This allowed for the discovery of mainly case reports containing heterogeneous information. This lack of data limited the statistical analysis as well as the construction of meta-analysis and subgroup variance analysis. Despite the rarity of malignant transformation of scar endometriosis, we still managed to isolate and portray evidence in the pathophysiology, investigation and management of the disease, as well as a summary of the diagnostic steps necessary and ideal surgical approach.

## 5. Conclusions

Malignant transformation of scar endometriosis is a rare complication. Endometrial implants in the abdominal wall should be considered as preventable complications of gynecological surgeries. Special attention should be paid to women with a history of cesarean section or uterine surgery or patients presenting with a palpable mass in the abdominal wall with or without symptoms. Future research should focus on surgical techniques and their improvement to avoid endometrial spillage.

## Figures and Tables

**Table 1:** Demographic features of reported cases.

Demographic	Median (Range)	Mean
Age at diagnosis (years)	47 (37–67)	48.01
Interval of AWE diagnosis since first surgery (years)	20 (4–41)	19.9
Time to recurrence (months)	9	13.56
Time to death (months)	19	37.67

**Table 2:** Clinicopathological features of 73 malignant transformation of abdominal wall endometriosis.

Clinicopathological Features	Case Numbers	(%)
**Lesion site**		
Cesarean section scar	61	(83.6%)
Other type of surgery	11	(15.1%)
Primary EMs	1	(1.4%)
**Main symptom**		
Mass	27	(37.0%)
Pain	27	(37.0%)
Both	14	(19.2%)
Others	3	(4.1%)
Unknown (NA)	2	(2.7%)
**Serum CA125**		
Negative	27	(37.0%)
Positive	18	(24.7%)
Unknown (NA)	28	(38.4%)
**Pathology**		
Clear cell carcinoma (CCC)	52	(71.2%)
Endometrioid carcinoma (EC)	11	(15.1%)
Mixed (CCC+EC or EC+SPC or EC+sarcoma)	6	(8.2%)
Serous adenocarcinoma (SA)	2	(2.7%)
Serous papillary carcinoma (SPC)	2	(2.7%)

CCC: Clear cell carcinoma, EC: endometrioid carcinoma, EMs: endometriosis, SA: serous adenocarcinoma, SPC: serous papillary carcinoma.

**Table 3:** Surgery of population with negative lymph nodes in preoperative assessment and postoperative histopathology results.

	**N (%)**	**N (%)**	**27**
**LNR**	**LNR +**	**LNR −**	**6**
	**3 (11.1)**	**3 (11.1)**	
LR + HRT + SOT + LNR (pelvic)		1	
LR + HRT + SOT + omentectomy + LNR (pelvic)		1	
LR + HRT + SOT + omentectomy + LNR (pelvic)		1	
LR + SOT + LNR (anterior abdominal wall)	1		
LR + HRT + SOT + LNR (left Pelvic)	1		
LR + HRT + SOT + LNR (Pelvic)	1		
No LNR			21
Surgery of population with positive lymph nodes in preoperative assessment and postoperative histopathology results			
	**N (%)**	**N (%)**	**20**
**LNR**	**LNR +**	**LNR −**	**17**
LR + HRT + SOT + omentectomy + LNR (pelvic)	1		
LR + HRT + SOT + omentectomy + LNR (inguinal, pelvic, para aortic)	1		
LR + HRT + SOT + omentectomy + LNR (inguinal, pelvic)	1		
LR + Curettage + LNR (inguinal, right pelvic)	1		
LR + HRT + SOT + omentectomy + LNR (inguinal, pelvic, para aortic)	1		
LR + HRT + SOT + LNR (inguinal, pelvic)	1		
LR + SOT + LNR (pelvic)	1		
LR + SOT + HRT + omentectomy + LNR (inguinal, pelvic, para aortic)	1		
LR + SOT + omentectomy + LNR (pelvic, para aortic)	1		
LR + HRT + SOT + LNR (pelvic right inguinal, para aortic)	1		
LR + HRT + SOT + omentectomy + LNR (inguinal, pelvic, para aortic)	1		
LR + HRT + SOT + LNR (pelvic)	1		
LR + HRT + SOT + LNR (inguinal, bilateral pelvic)		1	
LR + HRT + SOT + LNR (pelvic)	1		
LR + HRT + SOT + omentectomy + LNR (right inguinal, pelvic)	1		
LR + HRT + SOT + LNR (bilateral inguinal, pelvic, ileal, caecal)	1		
LR + HRT + SOT + LNR (8 AW)	1		
No LNR and/or lost in follow-up			3

LR: Local resection, HRT: total hysterectomy, SOT: salpingo-oophorectomy, LNR: lymph node resection, AW: abdominal wall.

**Table 4:** Surgical treatment strategy.

Surgery Type	Case Number	(%)
Yes	70	(100%)
LR	12	(17.1%)
LR + LNR	7	(10.0%)
LR + HRT + SOT	13	(18.6%)
LR + HRT + SOT + LNR	11	(15.7%)
LR + HRT + SOT + omentectomy + LNR	11	(15.7%)
LR + HRT + SOT + omentectomy	4	(5.7%)
Other	12	(17.1%)
No	2	
NA	1	

LR: Local resection, HRT: total hysterectomy, SOT: salpingo-oophorectomy, LNR: lymph node resection, NA: not available.

**Table 5:** Outcome in population according to lymph nodes from postoperative histopathology results.

	Positive Lymph Nodes		Negative Lymph Nodes		*p*-Value
	Case Number	(%)	Case Number	(%)	
Patients	22	(100%)	33	(100%)	
DOD	5	(22.7%)	4	(12.1%)	0.45
NED Progression	13 0	(59.1%)	18 1	(54.55%) (3.0%)	
Recurrence	1	(4.5%)	5	(15.1%)	
NA	3	(13.6%)	4	(12.1%)	

DOD: Died of disease; NED: no evidence of disease; NA: not available.

**Table 6:** Recurrence according to lymph node status.

	Case Number (%)				*p*-Value
	Preop positive lymph nodes		Preop negative lymph nodes		
Recurrence	8/20	(40.0%)	9/27	(33.3%)	0.64
	Postop positive lymph nodes		Postop negative lymph nodes		
Recurrence	7/22	(31.8%)	11/33	(33.4%)	0.84

## Supplementary Materials

The following supporting information can be downloaded at: https://www.mdpi.com/article/10.3390/jcm13082282/s1, Table S1. Summary of studies included in the review [5,11,12,14,17,21,24,25,26,27,34,64,65,66,67,68,69,70,71,72,73,74,75,76,77,78,79,80,81,82,83,84,85,86,87,88,89,90,91,92,93,94,95,96,97,98,99,100,101,102,103].
