# Supplementary material for: Malignancy in Abdominal Wall Endometriosis: Is There a Way to Avoid It? A Systematic Review
Source: J Clin Med. 2024 Apr 15;13(8):2282. doi: 10.3390/jcm13082282 (PMC11050881; doi:10.3390/jcm13082282)
Supplement: Supplementary file 1 [file jcm-13-02282-s001.zip › jcm-2945814-supplementary.pdf]

Supplementary Table S1. Summary of studies included in the review

ID	Authors, year	Age	Delay (years)	Previous gynecological surgery	Pre-existing endometriosis	Tumor histology	Symptoms	Tumor size (cm)	Diagnosis	IMAGING Appearance	Lymph nodes (pre-surgery)	CA125	Surgical resection extent	Lymph nodes (post-surgery)		Chemotherapy regimen	Radiation regimen	recurrence	Time to / location of recurrence	Time (months)	Follow-up (months)	Patient outcomes
1	Achach, 2008 [64]	49	20	Myomectomy	NA	CCC	Pain, Mass	8	CT	Heterogeneous mass	NA	NA	LR	Neg	None	Cyclophosphamide + Cisplatin	Yes	Yes	Bladder, pelvic bones		NA	Recurrence
2	Alberto, 2006 [43]	38	11	1 C/S + HRT, SOT for endometriosis	Yes	CCC	Pain	5	US, MRI	Cystic	NA	Normal	LR	Neg	None	Taxol + Carboplatin	Yes				NA	NA
3	Aust, 2015 [65]	47	16	1 C/S + HRT	No	CCC	Mass	10	CT, PET CT	NA	Pelvic	72	LR + SOT + LNR (pelvic and para-aortal lymphadenectomy) + omentactomy	Pos	2/48 lymph nodes +	Taxol + Carboplatin	No				10	NED
4	Bahall, 2022 [66]	57	29	2 C/S + 1 hysterectomy	Yes	CCC	Pain	15	CT	Heterogeneous mass	Pelvic	NA	LR + SOT + LNR (pelvic lymph node sampling)	Neg	None	Taxol + Carboplatin	No	Yes	Inguinal lymph nodes		NA	Recurrence
5	Bahall, 2022 [66]	46	10	1 laparoscopic ovarian drilling	Yes	CCC	Pain	10	CT	NA	AW	1200	LR + HRT + SOT + LNR (8 AW)	Pos	8/8 +	Taxol + Carboplatin	No				32	NED
6	Bats, 2008 [52]	38	13	1 C/S	Yes	CCC	Mass	11	CT, MRI	Solid Cystic	NA	NA	LR (2 mm margin) + HRT + SOT+ omentectomy + pelvic samples		/	Taxol + Carboplatin	No	Yes	Iliac lymph node		8	Recurrence
7	Behbehani, 2020 [67]	48	5	C/S + supracervical HRT + bladder resection in endometriosis treatment	Yes	CCC	Mass	6	MRI	Solid Cystic	NA	NA	LR (tumour margins neg) + bladder excision + SOT		/	Doxorubicine + Gemcitabine	No				NA	NA
8	Bedell, 2020 [31]	55	18	1 C/S	No	High grade Adenocarcinoma	Syncopal episode	6,5	CT, PET CT	Lobulated soft tissue mass	Inguinal + Pelvic	17,2	LR + rectus muscle + LNR (inguinal, pelvic, para.aortic) + HRT + SOT + omentectomy	Pos	2/2 right inguinal + , 1/14 pelvic+	Taxol + Carboplatin	Yes				8	NED
9	Bourdel, 2010 [58]	43	20	2 C/S + 1 endometriosis excision	Yes	CCC	Mass	9	MRI	Heterogeneous mass	Pelvic	43,8	LR + umbilicus +HRT + SOT + LNR bilat external Pelvic)	Pos	1 right Pelvic + , multiples left Pelvic +	Taxol + Carboplatin	Yes	Yes	6 / lymph nodes	6	22	DOD
10	Castagnino, 2021 [28]	49	20	1 C/S	No	CCC	Pain, Mass	25	MRI, PET CT	Polylobulated mass	Pelvic + Inguinal	49,8	LR (free margins) + HRT + SOT +LNR (bilateral Pelvic + inguinal)	Pos	bilateral Pelvic + inguinal+	Yes	No				NA	NA
11	Da Ines, 2010 [68]	48	20	2 C/S	Yes	EC + SPC	Pain	6	MRI	Solid Cystic Iso T1 W Hyper T2 W	None	Normal	LR + HRT + SOT + LNR (left Pelvic)	Pos	2/2 left Pelvic	Taxol + Carboplatin	No				15	NED
12	Dobrosz, 2014 [69],	42	NA	1 C/S	NA	CCC	Pain	NA	US	NA	None	NA	LR + enodmetrial biopsy	Neg	None	No	No				NA	NED
13	Drukala, 2010 [70]	43	17	1 C/S	NA	EC	Pain	NA	NA	NA	NA	High	LR + HRT	Neg	None	Cisplatin + Etoposide + 5-fluorouracil	Yes	Yes	48 / inguinal nodes	48	130	DOD
14	Fabregas, 2014 [71]	49	15	1 C/S + 1 endometriosis excision	Yes	SPC	Pain, Mass	7	US	NA	None	243	LR + HRT + SOT + LNR (Pelvic)	Pos	2 right + 1 left Pelvic	Taxol + Carboplatin	No				48	NED
15	Ferrandina, 2016 [24]	44	9	1 C/S	No	CCC	Abdominal bloating, Swelling	22	MRI, PET CT	Solid	Pelvic + Inguinal	Normal	LR + removal of rectus + HRT+ SOT + LNR (inguinal + pelvic)	Pos	7/14 pelvic, 8/11 inguinal	Taxol + Carboplatin	No	Yes	1 / NA	1	6	DOD
16	Ferrari, 2021 [72]	46	12	1 C/S, Resection of endometriomas laparoscopy, HRT + bilateral salpingectomy laparoscopy	Yes	CCC	Pain, Mass	5	US MRI	Hypo T1 W Hyper T2 W	NA	33	LR+ Bilateral Ovariectomy	Neg	None	No	No				4	NED

Supplementary Table S1. (continued)

ID	Authors, year	Age	Delay (years)	Previous gynecological surgery	Pre-existing endometriosis	Tumor histology	Symptoms	Tumor size (cm)	Diagnosis	IMAGING Appearance	Lymph nodes (pre-surgery)	CA125	Surgical resection extent	Lymph nodes (post-surgery)		Chemotherapy regimen	Radiation regimen	recurrence	Time to / location of recurrence	Time (months)	Follow-up (months)	Patient outcomes
17	Gentile, 2018 [101]	42	7	1 C/S + 1 local resection of endometriosis of the cephalic scar (Pfannenstiel)	Yes	CCC + EC	Pain, Mass	10	CT	Heterogeneous mass	NA	Normal	LR + LNR (inguinal + Pelvic)	Pos	1/8 Pelvic , 0/11 inguinal	Yes	NA				2	NED
18	Giannella, 2020 [25]	45	22	2 C/S	No	CCC	Pain, Mass	20	CT, MRI	Solid Lacunar aeras	Pelvic + Liver Pulmonary Metastasis	NA	None		Not evaluated	Taxol + Carboplatin after Gemcitabine	No	Yes	2 / lung, bones, local	2	7	DOD
19	Fodor, 2017 [73]	NA	NA	1 C/S	NA	CCC	Pain, Mass	5	US	Solid Cystic	Not evaluated	Normal	LR (no free margins, re-operated)	Neg	None	No	No				6	NED
20	Gücer, 1997 [99]	45	8	1 C/S + HRT	NA	EC	Pain	NA	NA		NA	NA	LR + ovaries	Neg	None	Carboplatine + Cyclophosfamide	Yes	NA	NA		20	DOD
21	Han, 2022 [74]	54	31	1 C/S + laparoscopy for endometrial cyst	Yes	EC	Mass	3	US, PET CT	Cystic	right Inguinal, Pelvic, retrodiaphragmatic retroperitoneal	48	LR and then HRT + SOT + LNR (pelvic)	Pos	right pelvic and inguinal+	Doxorubicin + Carboplatin	No				16	NED
22	Harry, 2007 [100]	55	30	Open sterilization	No	CCC	Pain	4	CT		Not evaluated	9	LR		Not evaluated	No	Yes				18	NED
23	Heller, 2014 [102]	37	NA	3 C/S	NA	CCC	Mass	NA	NA		NA	NA	LR + left SOT+ LNR (pelvic)	Pos	bilateral pelvic +	No	No				5	NA
24	Hitti, 1990 [21]	46	14	2 C/S	Yes	CCC	Pain	6	NA	NA	None	NA	LR + HRT + SOT + sigmoidectomy + colectomy	Neg	None	No	No				30	NED
25	Ishida, 2002[27]	56	20	2 C/S	NA	CCC	Mass	NA	CT	NA	Not evaluated	Normal	LR		Not evaluated	Cisplatin	No	Yes	Bone, brain, lung		24	DOD
26	Karpathiou, 2021 [75]	55	25	2 C/S	No	EC	Mass	6	US, PET CT	NA	None	NA	LR (free margins)	Neg	None	Carboplatin + Plaxitel	Yes				24	NED
27	Ji, 2017 [76]	48	15	1 C/S	Yes	Serous adenocarcinoma	Pain, Mass	15	CT, MRI, US	Hyper T1 W Hyper T2 W	inguinal	715	LR + SOT + HRT + omentectomy + LNR (pelvic+ para aortic + inguinal)	Pos	11/18 pelvic, 1/9 para aortic, 2/5 inguinal	Taxol + Carboplatin after Gemcitabine	No	NA	NA		7	Recurrence
28	Klein, 1999 [26]	67		no	No	CCC	Mass	16,5	CT, MRI	Heterogeneous mass, Hyper T2 W	None	Normal	LR + HRT+ SOT+ liver biopsy	Neg	None	No	Yes	Yes	25 / local recurrence	25	50	NED
29	Lai, 2019 [34]	52	19	1 C/S	No	CCC	Mass, Ulceration	17,5	CT	NA	None	20,1	LR(free margins 2 cm)+ HRT + SOT	Neg	None	No	No	Yes	10/ inguinal node, bone	10	14	Recurrence
30	Lai, 2019 [34]	56	33	1 C/S	No	CCC	Mass	6,5	CT	NA	Pelvic	22,3	LR (free margins 2 cm) + SOT+ HRT+ omentectomy+ LNR (pelvic)	Pos	right pelvic	Taxol + Carboplatin	No	Yes	3 / inguinal node	3	11	NED
31	Lai, 2019 [34]	56	24	1 C/S	No	CCC	Mass	12	CT	Heterogeneous mass	None	23	LR (free margins 2cm) + HRT + SOT + LNR (pelvic)	Neg	None	Taxol + Carboplatin	No				5	NED
32	Lai, 2019 [347]	55	24	1 C/S	Yes	CCC	Mass	12,5	CT	NA	None	26,7	LR (no free margins) + HRT + SOT + omentectomy	Neg	None	Gemcitabine + Carboplatin + Bavacizumab	No	Yes	Abdominal wall, inguinal node, neck node, liver, lung		23	DOD
33	Lai, 2019 [34]	45	20	1 C/S	No	CCC	Mass, Ulceration	4,8	CT	NA	None	38,9	None	Neg	None	Taxol + Carboplatin after Doxorubicin + Carboplatin	Yes	Yes	Abdominal wall, inguinal node		7	Progression

ID	Authors, year	Age	Delay (years)	Previous gynecological surgery	Pre-existing endometriosis	Tumor histology	Symptoms	Tumor size (cm)	Diagnosis	IMAGING Appearance	Lymph nodes (pre-surgery)	CA125	Surgical resection extent	Lymph nodes (post-surgery)		Chemotherapy regimen	Radiation regimen	recurrence	Time to / location of recurrence	Time (months)	Follow-up (months)	Patient outcomes
34	Lai, 2019 [34]	52	4	Left oophorectomy	Yes	CCC	Mass	7	CT	NA	None	38,5	LR (free margins 2cm) + HRT + SOT	Neg	None	Taxol + Carboplatin	No				97	NED
35	Leng, 2006 [11]	41	16	1 C/S + 3 endometriosis resection	Yes	Sarcoma + EC	Pain	10	Biopsy	Solid Cystic	NA	NA	LR (5mm free margin) + HRT + SOT	Neg	None	Cisplatin + Ifosfamide	No	Yes	Local recurrence		15	DOD

Supplementary Table S1. (continued)

ID	Authors, year	Age	Delay (years)	Previous gynecological surgery	Pre-existing endometriosis	Tumor histology	Symptoms	Tumor size (cm)	Diagnosis	IMAGING Appearance	Lymph nodes (pre-surgery)	CA125	Surgical resection extent	Lymph nodes (post-surgery)		Chemotherapy regimen	Radiation regimen	recurrence	Time to / location of recurrence	Time (months)	Follow-up (months)	Patient outcomes
36	Lewis, 2022 [77]	54	22	1 C/S	Yes	CCC	Pain, Mass	6	CT, MRI, US	NA	Pelvic + Inguinal	45	LR (neg margins) + HRT + SOT + omentectomy + LNR(right inguinal + pelvic + Pelvic)	Pos	2/4 right inguinal +2/5(obturator) pelvic +0/5 Pelvic	Carboplatin + Plaxitel + trastuzumab	No				9	NED
37	Li, 2019 [59]	49	26	1 C/S, cholecystectomie, splenectomy	No	CCC	Pain	8,2	CT	NA	None	57,1	LR + SOT + HRT	Neg	None	Taxol + Carboplatin	No				8	NED
38	Ijichi, 2014 [57]	60	35	2 C/S	NA	CCC	Mass	4	MRI	Solid Cystic	None	Normal	LR		Not evaluated	No	No	Yes	c/s scar		8	Recurrence
39	Liu, 2021 [14]	48	22	1 C/S	Yes	CCC	Pain, Mass	13	CT, MRI, Biopsy	Cystic, solid Hypo T1 W Hyper T2 W	None	164,7	LR + SOT + HRT	Neg	None	Cisplatin	Yes				12	NED
40	Fargas, 2014 [71]	39	20	1 C/S	Yes	CCC	Pain, Mass	6	CT	NA	Inguinal	22,1	LR + bladder excision + LRT + SOT + omentectomy + LNR (pelvic , para aort , inguinal)	Pos	8/8 inguinal, 18/21 pelvic, 6/6 para aortic	Taxol + Carboplatin	No	Yes	Local recurrence		12	DOD
41	Lopes, 2019 [78]	48	30	1 C/S	Yes	CCC	Mass	12	CT, MRI	Solid	Pelvic + inguinal	3157	LR + HRT + SOT + LNR (inguinal, pelvic , para aortic) + omentectomy	Pos	2/9 inguinal, 2/20 right pelvic 1/24 left pelvic	NA	NA				NA	NED
42	Markopoulos, 1996 [103]	40	25	2 C/S	No	EC	Pain	NA	NA		NA	NA	HRT + SOT		Not evaluated	No	No				24	NED
43	Marques, 2017 [79]	47	30	3 C/S	NA	CCC	Pain, Mass	8	CT	Heterogeneous mass	None	29	LR (free margin) + SOT after: HRT	Neg	None	Taxol + Carboplatin	No	Yes	9 / local	9	36	NED
44	Matsuo, 2008 [5]	37	NA	Laparotomy for endometrioma resection	Yes	CCC	Pain, Abdominal bloating, Swelling	14	MRI	Cystic	None	NA	LR + HRT + SOT+ LNR (pelvic) + omentectomy	Neg	None	Docetaxel + Carboplatin	No	Yes	18 / local recurrence + bowel	18	18	Recurrence
45	Matter, 2003 [12]	60	41	2 C/S	No	EC	Pain	8	CT, MRI	NA	None	Normal	LR	Neg	None	No	No				18	NED
46	Mert, 2012 [80]	42	NA	2 C/S+ tubal ligation	NA	CCC	Mass	17	CT	NA	None	Normal	LR + HRT + SOT + omentectomy + LNR (pelvic)	Neg	None	Taxol + Carboplatin	No				26	NED
47	Mert, 2012 [80]	51	18	2 C/S + HRT	Yes	CCC	Mass	7	CT	NA	NA	NA	LR + SOT + omentectomy	Neg	None	No	Yes				31	NED
48	Mihailovici, 2017 [9]	NA	NA	1 C/S	NA	CCC	Pain, Abdominal bloating, Swelling	9	CT, US, PET CT	Solid Heterogeneous mass	Inguinal	Normal	LR + SOT + HRT		/	Platinium	Yes				NA	NA
49	Miller, 1997 [81]	38	9	1 C/S	Yes	CCC	Pain	4	CT, US	NA	None	35	LR, then HRT + SOT + omentum	Neg	None	Cisplatin	Yes				60	NED
50	Obata, 2013 [82]	60	12	HRT	No	CCC	Bleeding	3	MRI, PET CT	NA	None	NA	LR, SOT	Neg	None	NA	NA				NA	NA
51	Omranipour, 2010 [29]	59	20	Laparotomy for perforation during curettage	No	SPC	Mass	10	CT	Solid Cystic	None	Normal	LR + SOT + HRT	Neg	None	Platinium	Yes				24	NED
52	Park, 1999 [83]	54	26	2 C/S	Yes	CCC	Mass	5	CT	NA	not evaluated	NA	LR		Not evaluated	No	Yes				NA	NA
53	Paulino, 2020 [84]	45	23	Hemicolectomy	No	EC	Pain	18	CT	NA	Inguinal	NA	LR (free margins) + HRT + SOT	Pos	inguinal and axillairy confirmed by biopsy	No	No				12	NA
54	Peer, 2013 [10]	44	NA	Appendicectomy	NA	EC	Mass	3	CT, MRI, PET CT	Cystic	Inguinal	Normal	LR + LNR (right pelvic + inguinal) + curettage	Pos	0/12	Taxol + Carboplatin	Yes				36	NED
55	Petit, 2022 [85]	52	NA	Resection parietal endometriosis	Yes	CCC	Pain	8	US, MRI, PET CT	Cystic	None	NA	LR + SOT + HRT	Neg	None	Carboplatin-Taxol-Avastin)	No					NA

				nodule, small bowel resection, 2 C/S																		
56	Provendier, 2020 [86]	44	14	2 C/S	No	CCC	Mass	6	US, MRI	Solid mass Hypo T1 W Hyper T2 W	None	NA	LR + SOT + HRT	Neg	None	No	Yes				5	NED
57	Razzouk, 2007 [87]	46	26	2 C/S + 2 endometriosis resection	Yes	CCC + EC	Mass	20	MRI	Heterogeneous mass, Hyper T2 W	None	107	LR + SOT + LNR (anterior abdominal wall)	Pos	AW 1/1	Taxol + Carboplatin	No	Yes	Liver, lymph nodes		6	DOD

Supplementary Table S1. (continued)

ID	Authors, year	Age	Delay (years)	Previous gynecological surgery	Pre-existing endometriosis	Tumor histology	Symptoms	Tumor size (cm)	Diagnosis	IMAGING Appearance	Lymph nodes (pre-surgery)	CA125	Surgical resection extent	Lymph nodes (post-surgery)		Chemotherapy regimen	Radiation regimen	recurrence	Time to / location of recurrence	Time (months)	Follow-up (months)	Patient outcomes
59	Ruiz, 2015 [89]	41	20	1 C/S	No	CCC	Pain, Bleeding	14,8	CT, US, MRI	Heterogeneous mass	None	22	LR + HRT+ SOT+ omentectomy	Neg	None	Taxol + Carboplatin	Yes	Yes	c/s scar		6	Recurrence
60	Ruiz, 2015 [89]	57	30	3 C/S	No	CCC + EC	Pain	19,4	CT, MRI	NA	inguinal	81	LR + HRT+ SOT + LNR (bilateral inguinal + Pelvic, + ileal + caecal)	Pos	1 inguinal, 1 internal Pelvic	Taxol + Carboplatin	Yes				NA	NED
61	Rust, 2008 [90]	42	NA	HRT	NA	CCC	Pain	5	NA	NA	Not evaluated	NA	LR		Not evaluated	No	No				NA	NA
62	Sawazaki, 2012 [91]	41	18	2 C/S	NA	CCC	Pain	4,8	MRI	NA	NA	NA	LR+ partial bladder, partial rectus		Not evaluated	Taxol + Carboplatin	No				4	NA
63	Schnieber, 1986 [22]	40	15	1 C/S	Yes	CCC	NA	NA	NA		NA	NA	HRT + SOT			No	Yes	NA	NA		18	DOD
64	Sergent, 2006 [92]	45	28	2 C/S	No	CCC	Mass	5	MRI	Heterogeneous Hypo T1 W Hyper T2 W	NA	NA	LR + SOT + HRT		NA	Taxol + Carboplatin	No	Yes	Local recurrence		6	DOD
65	Shalin, 2012 [93]	47	NA	1 C/S	Yes	CCC	Pain, Ulceration	6	MRI	Lobular enhancing lesion	NA	Normal	LR+ ovarian cyst, endometrium + LNR (Pelvic)	Pos	2/4 Pelvic	Platinium	Yes				7	NED
66	Stevens, 2013 [98]	51	NA	1 C/S	NA	EC	Mass	NA	NA		NA	NA	LR + HRT + SOT + omentectomy + LNR (pelvic)	Neg	None	Yes	Yes				6	NED
67	Taburiaux, 2015 [17]	56	29	1 C/S	Yes	EC	NA	8	NA		NA	NA	NA		NA	Taxol + Carboplatin	No				17	NED
68	Tsuruga, 2019 [94]	49	15	Laparoscopy for endometrioma	Yes	CCC + EC	Pain, Mass	4	US, MRI, PET CT	Solid Cystic Hypo T1 W Hyper T2 W	None	90	LR +HRT +SOT + omental biopsy	Neg	None	Taxol + Carboplatin	No				NA	NED
69	Vinchant, 2013 [95]	43	19	2 C/S	Yes	EC	Pain	13	CT, MRI, PET CT	Solid Cystic	Pelvic + inguinal	NA	LR after HRT + SOT + LNR (pelvic right inguinal para aortic)		NA	Caelyx-Carpoplatin neo adjuvant	No	NA	NA		26	NA
70	Wang, 2020 [96]	43	19	1 C/S	NA	CCC	Mass	10	CT, Biopsy, MRI		NA	26,2	HRT + LNR + LR +SOT			Taxol + Carboplatin	NA				NA	NA
71	Wei, 2016 [97]	46	18	1 C/S	NA	CCC	Pain, Ulceration	7	CT	Solid Cystic	Not evaluated	NA	LR + SOT + HRT		Not evaluated	No	No				NA	NA
72	Williams, 2009 [101]	53	17	1 C/S	No	CCC	Pain, Mass	5	MRI	NA	Pelvic	39	LR + HRT + SOT + omentectomy + LNR (pelvic + inguinal)	Pos	10/14 pelvic, 17/17 inguinal	Taxol + Carboplatin	No	yes	Mediastinal nodes		11	DOD
73	Yan, 2011 [47]	41	NA	2 C/S + 1 endometriosis excision	Yes	CCC	Pain	10	CT	Lobulated	not evaluated	6,3	LR		Not evaluated	Yes	No				24	NED

SOT, Salpingo-oophorectomy; HRT, Hysterectomy; C/S, Cesarean-section; CCC, Clear cell carcinoma; SPC, Serous papillary carcinoma; EC, Endometrioide carcinoma ; CT, Computed tomography; MRI, Magnetic resonance imaging; US, Ultrasonography; LR, Local resection of the mass; LNR, Lymph nodes resection; DOD, Death of disease; NED, No evidence of disease; NA, Non available.
